# Abrogation of collagen-induced arthritis by a peptidyl arginine deiminase inhibitor is associated with modulation of T cell-mediated immune responses

**DOI:** 10.1038/srep26430

**Published:** 2016-05-23

**Authors:** Joanna Kawalkowska, Anne-Marie Quirke, Fatemeh Ghari, Simon Davis, Venkataraman Subramanian, Paul R. Thompson, Richard O. Williams, Roman Fischer, Nicholas B. La Thangue, Patrick J. Venables

**Affiliations:** 1 Kennedy Institute, Nuffield Department of Orthopaedics, Rheumatology & Musculoskeletal Sciences, University of Oxford, Roosevelt Drive, Oxford OX3 7FY, UK; 2 Laboratory of Cancer Biology, Department of Oncology, University of Oxford, Old Road Campus Research Building, Roosevelt Drive, Oxford OX3 7DQ, UK; 3 Target Discovery Institute, Nuffield Department of Medicine, University of Oxford, Roosevelt Drive, Oxford OX3 7FZ, UK; 4 Biochemistry and Molecular Pharmacology, University of Massachusetts Medical School, LRB 826, 364 Plantation Street, Worcester, MA, 01605, USA

## Abstract

Proteins containing citrulline, a post-translational modification of arginine, are generated by peptidyl arginine deiminases (PAD). Citrullinated proteins have pro-inflammatory effects in both innate and adaptive immune responses. Here, we examine the therapeutic effects in collagen-induced arthritis of the second generation PAD inhibitor, BB-Cl-amidine. Treatment after disease onset resulted in the reversal of clinical and histological changes of arthritis, associated with a marked reduction in citrullinated proteins in lymph nodes. There was little overall change in antibodies to collagen or antibodies to citrullinated peptides, but a shift from pro-inflammatory Th1 and Th17-type responses to pro-resolution Th2-type responses was demonstrated by serum cytokines and antibody subtypes. In lymph node cells from the arthritic mice treated with BB-Cl-amidine, there was a decrease in total cell numbers but an increase in the proportion of Th2 cells. BB-Cl-amidine had a pro-apoptotic effect on all Th subsets *in vitro* with Th17 cells appearing to be the most sensitive. We suggest that these immunoregulatory effects of PAD inhibition in CIA are complex, but primarily mediated by transcriptional regulation. We suggest that targeting PADs is a promising strategy for the treatment of chronic inflammatory disease.

Citrullination is a post-translational modification (PTM) of arginine, catalysed by peptidyl arginine deiminases (PADs) and may be important in generating autoantibodies to citrullinated proteins in rheumatoid arthritis (RA). Citrullination can also be pathogenic by modulating transcription of cytokines and generation of pro-inflammatory extracellular proteins (reviewed in Wegner *et al*.^1^). There are 5 different PAD enzymes, with PAD2 and PAD4 being the main isoforms involved in arthritis^1^. Traditionally PAD4 was thought to be the more important, as a polymorphism of the *PADI4* gene is associated with the prevalence of RA, but mainly in Asian populations^2^. Furthermore, PAD4 was thought to be the only PAD which could localize to the nucleus and, therefore, be involved in transcriptional regulation^2^,^3^. However more recent studies have highlighted the relative importance of PAD2 by showing it to be up-regulated in the inflamed joint^4^ and by demonstrating that like PAD4, it could translocate to the nucleus and have a specific role in the citrullination of histone H3^5^.

To examine the potential for PAD inhibition in the treatment of inflammatory disease, we chose collagen-induced arthritis (CIA) as a robust and reproducible model of RA^6^. We used the second generation pan PAD inhibitor BB-Cl-amidine (BB-Cl) which is equipotent against PAD4 as its precursor drug, Cl-amidine, but 10 times more potent against PAD2^7^. BB-Cl-amidine retains the critical elements of Cl-amidine but has a C-terminal benzimidazole and N-terminal biphenyl moiety (the “BB” in its nomenclature), which increases its plasma half-life and facilitates cellular uptake. In previous studies, the PAD inhibitor Cl-amidine was shown to have a modest anti-inflammatory effect, when given prophylactically at high doses^8^. In the current study, we use a therapeutic, rather than prophylactic, treatment protocol, which is more relevant for translation into human disease.

Here we demonstrate that BB-Cl-amidine reverses immune-mediated joint inflammation in a pre-clinical mouse model of arthritis. By targeting PAD enzymes, BB-CL-amidine reduces citrullination which is induced during inflammatory conditions such as arthritis. In addition, BB-CL-amidine-treatment decreases Th1 and Th17 responses *in vivo* while conversely, Th2 responses are supported. Thus, we report a novel treatment for immune-mediated pathologies in which the balance between Th17 and Th2 cells is disturbed.

## Results

### BB-Cl-amidine reduces inflammation and joint destruction in arthritic mice

**Figure 1:**
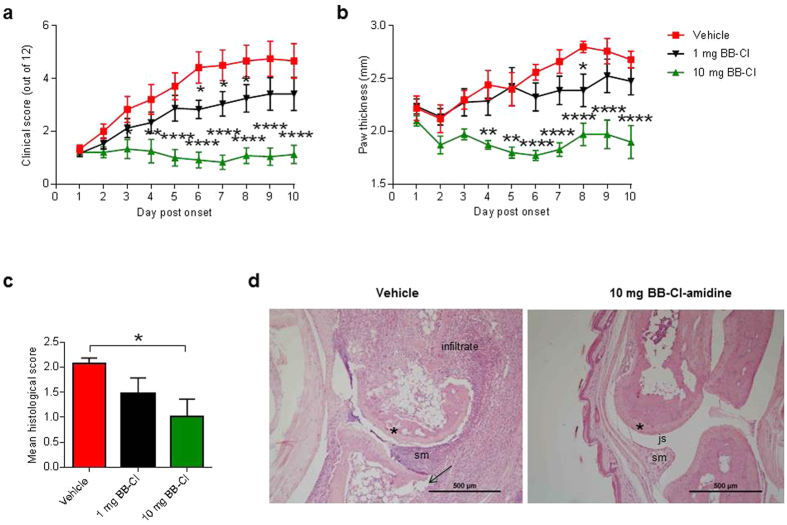
BB-Cl-amidine ameliorates severity of CIA. The severity of disease was evaluated daily by (**a**) clinical score and (**b**) paw swelling of the first hind paw to develop arthritis. Pooled data of the mean and SEM from two independent CIA experiments are shown (n = 12–13 animals per group). *P < 0.05; **P < 0.01; ****P < 0.0001. Two-way analysis of variance (ANOVA) with Tukey’s multiple comparisons test. (**c**) Mean histological scores (n = 4–5 per group). Error bars represent ± SEM (*P < 0.05). One-way analysis of variance (ANOVA) with Dunnett’s multiple comparisons test relative to vehicle-treated mice. (**d**) Representative images of the metatarsal-phalangeal joint of a vehicle or BB-Cl-amidine 10 mg/kg/day treated mouse are shown. In the vehicle-treated mice, there was a marked inflammatory infiltrate, bone erosion (arrows), destruction of articular cartilage (*), and proliferation of synovial membrane (sm). In the 10 mg/kg BB-Cl-amidine treated mice the cartilage (*) and joint space (js) were preserved, but there were still minor proliferative changes in the synovial membrane (sm).

To examine the therapeutic potential of BB-Cl-amidine *in vivo* we used the drug in a treatment protocol, that is, after the onset of arthritis. Compared with vehicle-treated mice, there was reduced clinical scoring (*P *< 0.0001 from 5 days treatment), paw swelling (*P *< 0.0001 from 6 days treatment), with a partial response in all measures at 1 mg/kg (Fig. 1a,b).

These clinical changes were reflected in a significant reduction of the histology scores (Fig. 1c). The histological changes in the vehicle treated group were typical of that seen in severe CIA (intense inflammatory cell infiltration and severe erosive changes) and almost completely normalised in the 10 mg/kg treatment group (Fig. 1d). During the 9 days of treatment, BB-Cl-amidine did not induce any adverse effects in the arthritic mice such as excessive weight loss (data not shown).

### BB-Cl-amidine reduces protein citrullination *in vivo* without affecting the ACPA response

**Figure 2:**
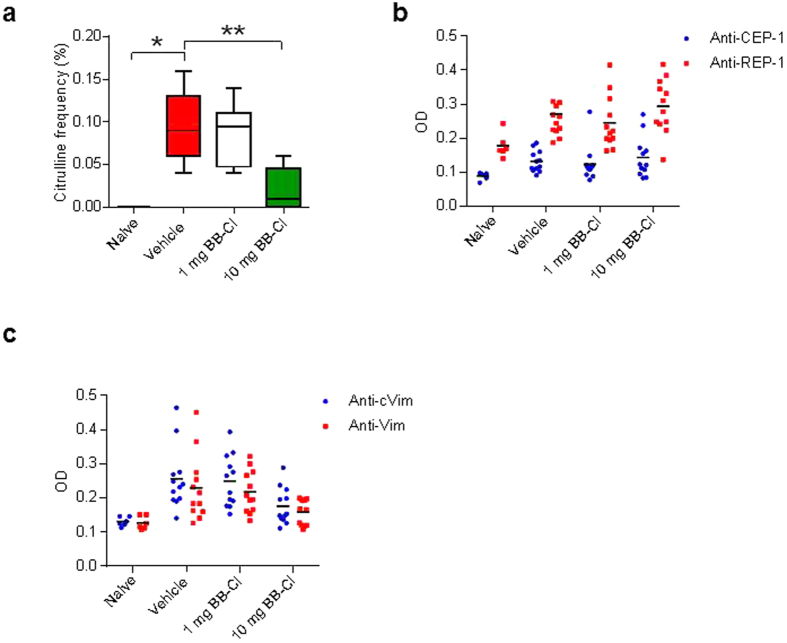
BB-Cl-amidine targets protein citrullination *in vivo* with little effect on immune responses against citrullinated antigens. (**a**) BB-Cl-amidine treatment of arthritic mice lead to a significant decline in the level of global protein citrullination in the lymph nodes as detected by mass spectrometry (n = 5–6 animals per group). *P < 0.05; **P < 0.01. Citrulline frequency is calculated as percentage of validated and citrullinated peptide sequences/identified peptides sequences at 1% false discovery rate. Error bars represent ± SEM. One-way analysis of variance (ANOVA) with Dunnett’s multiple comparisons test relative to vehicle-treated animals (control). (**b**,**c**) There were no differences between treatment groups in IgG immune responses against citrullinated α-enolase (CEP-1) and vimentin (cVim) versus their arginine-bearing controls. Each data point represents one animal. OD, optical density.

To confirm that treatment with BB-Cl-amidine reduced protein citrullination *in vivo*, we compared inguinal lymph nodes from arthritic mice treated with BB-Cl-amidine with vehicle-treated controls using mass spectrometry. We chose lymph nodes for study, as opposed to joints, because of our interest in the possible immunoregulatory effects of citrullination. No citrulline residues were detected in lymph nodes from naïve mice whereas the frequency of detected citrulline residues was increased in the vehicle-treated mice with arthritis. The frequency of detected citrulline residues was reduced by approximately 90% in the 10 mg/kg group with no change in the 1 mg/kg group (Fig. 2a). The detected citrulline residues were confirmed in every case by visual examination of the spectra. Individual citrullinated residues were detected in 34 different proteins (Supplementary Table 1). Noteworthy amongst these was R93 from histone H4, and R117 from histone H3.2. To examine the anti-citrullinated protein antibody (ACPA) response in the mice, we chose two well-characterised peptides from citrullinated alpha-enolase (CEP-1) and from citrullinated vimentin (cVim), on the basis that both sequences were highly conserved between mice and humans, both had arginine-containing controls, and both had been previously demonstrated to give robust and reproducible results in murine systems^9^,^10^. Both anti-CEP-1 and anti-cVim was increased in the collagen immunised mice, but in neither case was the response citrulline-specific, as illustrated by binding to the control peptides (REP-1 and Vim) (Fig. 2b,c). This confirmed a lack of a true ACPA response in CIA mice, which was not affected by BB-Cl-amidine treatment.

### BB-Cl-amidine treatment of arthritic mice is associated with Th2-type responses

**Figure 3:**
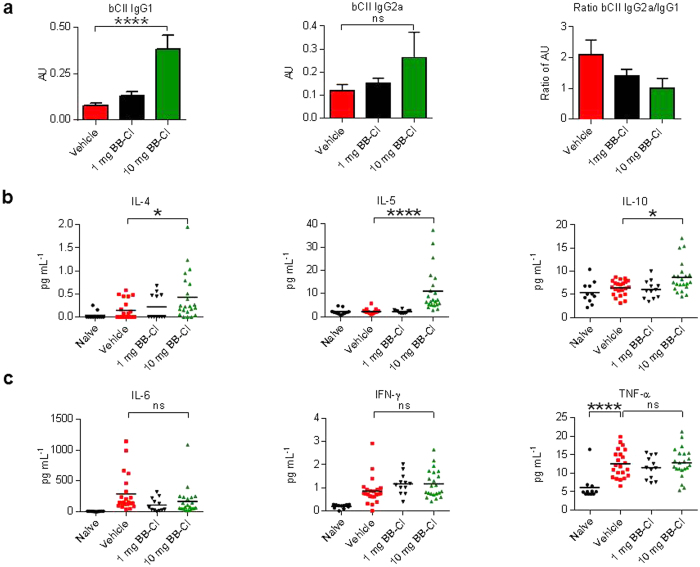
Analysis of IgG subclass anti-collagen antibodies and serum cytokines indicates an induction of a Th2 response by treatment with BB-Cl-amidine. (**a–c**) Pooled data from 2–3 independent CIA experiments are shown (n = 12–22 animals per group). (**a**) There was a significant rise in IgG1 anti–bovine collagen type II (bCII) antibodies in the 10 mg/kg BB-Cl-amidine treated group, with relatively little change in IgG2a anti-bCII. There was a fall in the IgG2a/IgG1 ratio with increasing BB-Cl-amidine treatment indicating induction of a Th2 response. AU, arbitrary units. (**b**) Increased serum levels of the Th2 cytokines IL-5 and IL-10. (**c**) There was no change in the pro-inflammatory cytokines IL-6, interferon-γ and TNF-α. (**b,c**) Each data point represents one animal. (**a–c**) Error bars represent ± SEM. One-way analysis of variance (ANOVA) with Dunnett’s multiple comparisons test relative to vehicle-treated animals. *P < 0.05; ***P < 0.001; ****P < 0.0001; ns, not significant.

Having demonstrated that the profound therapeutic benefit of BB-Cl-amidine in CIA was unlikely to be due to modulation of an ACPA response, we then went on to examine other possible immunological mechanisms. The level of anti-bovine type II collagen IgG1 was significantly increased in the mice treated with BB-Cl-amidine at 10 mg/kg. There was also a marginal increase in IgG2a anti-collagen antibodies but an overall fall in the ratio of the IgG2a/IgG1 in proportion to the dose of BB-Cl-amidine, indicating the possibility of a switch to Th2-type response induced by the drug (Fig. 3a). This was confirmed by the fact that serum levels of the Th2-type cytokines, IL-4, IL-5 and IL-10, were raised in response to treatment with BB-Cl-amidine, with IL-5 and IL-10 levels reaching statistical significance in the higher treatment dose (Fig. 3b). Levels of IL-6, TNF-α, IFN-γ, IL-1β, IL-2 and IL-17A were unchanged (Fig. 3c and data not shown).

### BB-Cl-amidine modulates the CD4^+^ T cell cytokine expression in arthritic mice and their proliferative capacity *ex vivo*

**Figure 4:**
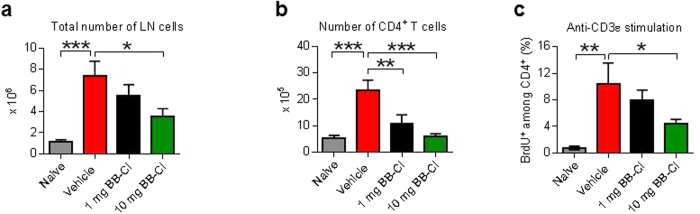
BB-Cl-amidine restrains T cell numbers and proliferation in inguinal lymph nodes from mice with CIA. (**a–c**) Data from CIA experiment is shown (n = 7 animals per group). (**a**) The total number of cells in the inguinal lymph nodes on day 10 was decreased with BB-Cl-amidine treatment. (**b**) The total number of CD4^+^ T cells in the inguinal lymph nodes of CIA mice is significantly lower in BB-Cl-amidine-treated mice. (**c**) BB-Cl-amidine decreases the percentage of proliferating CD4^+^ T cells (CD4^+^ BrdU^+^) in response to anti-CD3 antibody stimulation *ex vivo*. (**a–c**) Error bars represent ± SEM. One-way analysis of variance (ANOVA) with Dunnett’s multiple comparisons test relative to vehicle-treated animals. *P < 0.05; **P < 0.01; ***P < 0.001; ns, not significant.

To examine responses of T cells more directly, we examined T cells *ex vivo* taken from inguinal lymph nodes at day 10 after disease onset from each of the groups of arthritic mice. Compared to naive mice, there was an increase in numbers of total cells and CD4^+^ T cells in the vehicle treated group, which fell in response to BB-Cl-amidine treatment (Fig. 4a,b). There was a marked increase in the proliferative response of lymph node T cells to anti-CD3 stimulation in the vehicle-treated mice with CIA (Fig. 4c), which was significantly reduced in T cells taken from mice treated with the higher dose of BB-Cl-amidine, indicating an immunoregulatory or immunosuppressive effect of the drug.

**Figure 5:**
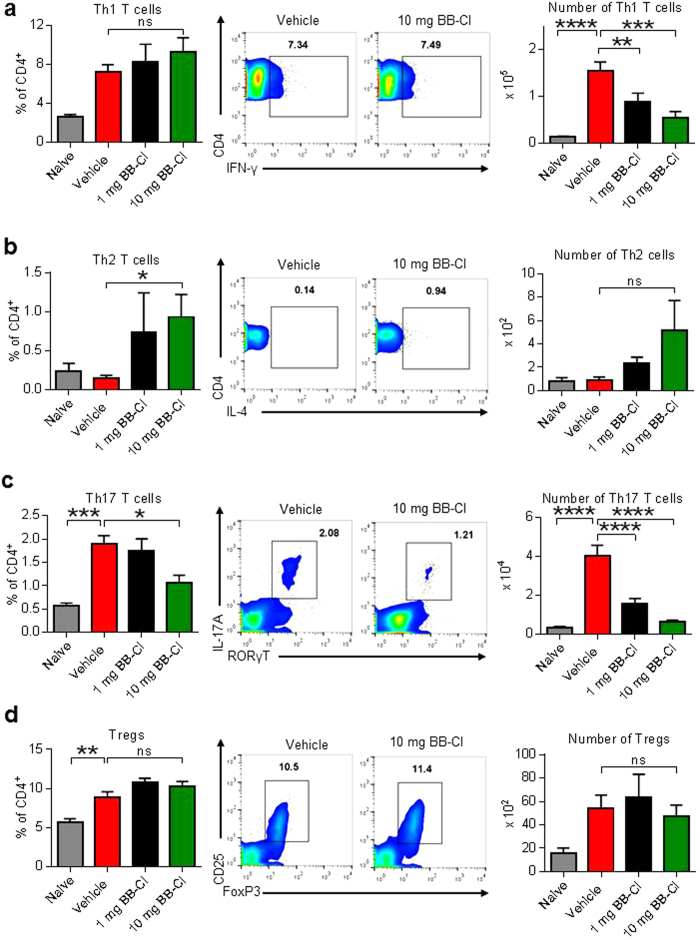
BB-Cl-amidine modulates the cytokine profile of T helper cell subsets, but not Tregs in the lymph nodes of arthritic mice. (**a–d**) Arthritic mice were culled on day 10 and the therapeutic effect of BB-CL-amidine on CD4^+^ T cells in lymph nodes was determined by flow cytometry (n = 7). BB-Cl-amidine decreases the frequency of (**a**) IFN-γ and (**b**) IL-17A producing cells whereas (**c**) IL-4 producing cells are increased. One representative FACS plot for each cell type is shown. Numbers in plots indicate the percent of cytokine secreting cells gated on live CD4^+^ T cells. RORγT, retinoic acid receptor-related orphan nuclear receptor gamma. (**d**) BB-Cl-amidine treatment does not alter the number of Tregs in the lymph nodes of mice with CIA. The percentage of CD25^+^ FoxP3^+^ cells among CD4^+^ T cells as well as the total number of Tregs in the lymph nodes is shown. One representative dot plot is shown. Numbers in plots indicate the percent of CD25^+^ FoxP3^+^ cells among live CD4^+^ T cells. (**a**–**d**) Error bars represent ± SEM. One-way analysis of variance (ANOVA) with Dunnett’s multiple comparisons test relative to vehicle-treated animals. *P < 0.05; **P < 0.01; ***P < 0.001; ****P < 0.0001; ns, not significant.

To dissect the mechanisms of BB-Cl-amidine further, we then quantified T helper cell subsets in the lymph nodes of arthritic mice on day 10. There was a fall in the total number of pro-inflammatory Th1 cells, but no change in the percentage of cells producing IFN-γ (Fig. 5a). In contrast, there was a rise in the percentage of Th2 cells (CD4 and IL-4 positive), reaching statistical significance at the 10 mg/kg dose of BB-Cl-amidine (Fig. 5b). These findings confirm and consolidate the increase Th2/Th1 ratio inferred by our findings with the IgG subclasses and cytokine profiles (Fig. 3). In our study, we found a marked suppression of Th17 cells by BB-Cl-amidine *ex-vivo* expressed both as total cell numbers and as percentages of CD4 T cells (Fig. 5c). In the mice with CIA there was an increase in Treg numbers compared to naïve animals, but importantly no effect from treatment with BB-Cl-amidine at either dose (Fig. 5d).

### BB-Cl-amidine has a direct effect on cytokine secretion and survival of CD4^+^ T helper subsets

**Figure 6:**
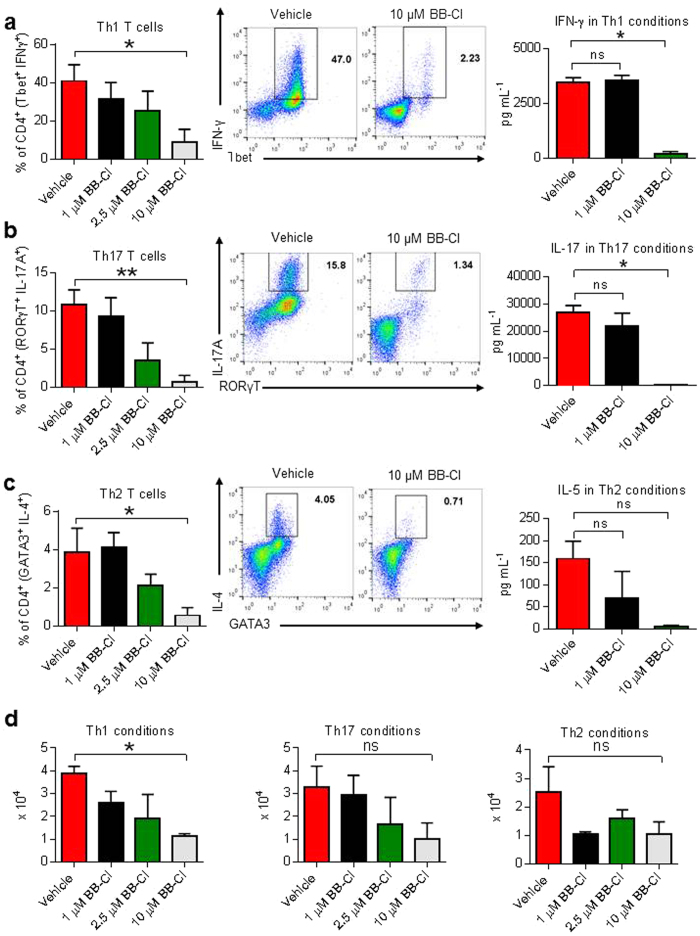
BB-Cl-amidine inhibits the induction of Th1 and Th17 cells and reduces their numbers *in vitro.* (**a–d**) Mouse CD4^+^ T cells were cultured in Th1, Th2 or Th17 differentiating conditions with BB-Cl-amidine or vehicle (DMSO) at 1 μM, 2.5 μM or 10 μM. After 5 days cell culture supernatants were investigated for cytokines, the cells were counted and analysed by flow cytometry. Culturing CD4^+^ T cells with BB-Cl-amidine decreases the amount of (**a**) IFN-γ, (**b**) IL-17A and (**c**) IL-4. (**d**) Culturing CD4^+^ T cells with BB-Cl-amidine decreases the total number of cells in Th1 conditions. (**a–d**) Shown is pooled data from 3 independent experiments with 3 biological repeats per experiment (n = 9 per group). Error bars represent ± SEM. One-way analysis of variance (ANOVA) with Dunnett’s multiple comparisons test relative to vehicle (control). *P < 0.05; **P < 0.01; ns, not significant.

To determine whether the suppression of T helper responses was a direct effect of BB-Cl-amidine, we cultured naive CD4^+^ T cells from healthy, unimmunised mice under Th1, Th17 or Th2 promoting conditions for 5 days with BB-Cl-amidine or vehicle added at the start of the culture. BB-Cl-amidine at 10 μM inhibited the induction of Th1, Th17 cells and in contrast to the *ex-vivo* findings, also Th2 cell induction (Fig. 6a–c). Similarly, analysis of day 5 culture supernatants revealed that BB-Cl-amidine at 10 μM suppressed IFN-γ and IL-17A secretion. Interleukin-4 levels were apparently unchanged, but this could be explained by the addition of extrinsic IL-4 to the cultures as part of the Th2 induction protocol (data not shown). However when IL-5 levels were measured as an independent Th2 cytokine, levels fell to the same extent as seen with IFN-γ and IL-17A (Fig. 6c). In addition, the number of total cells in Th1 conditions was reduced after co-culture with BB-Cl-amidine for 5 days, suggesting that citrullination is an important factor in the proliferation and survival of at least some T helper cell populations (Fig. 6d).

**Figure 7:**
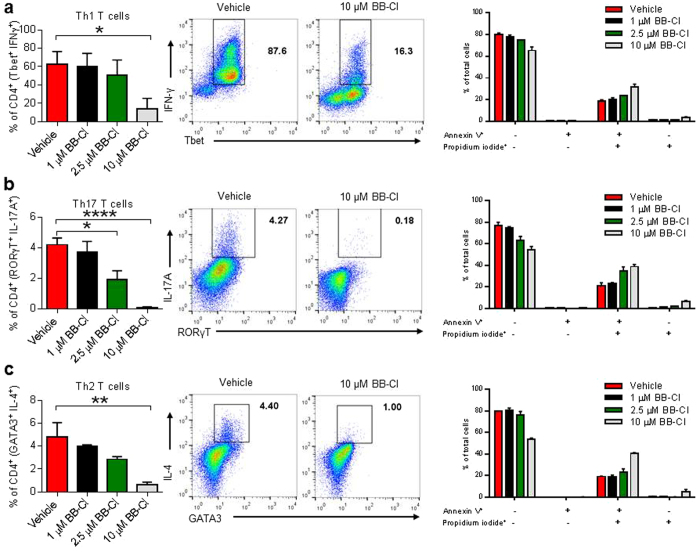
BB-Cl-amidine induces apoptosis and modulates the cytokine profile of CD4^+^ T helper cells *in vitro.* (**a–c**) Mouse naïve CD4^+^ T cells were cultured in Th1, Th2 or Th17 differentiating conditions. Vehicle (DMSO) or BB-Cl-amidine were added to the last 20 hours of culture. After 5 days the cells were analysed by flow cytometry to detect cytokines or annexin V and propidium iodide-positive cells. Adding BB-Cl-amidine to cultured T helper cells decreases the amount of (**a**) IFN-γ, (**b**) IL-17A and (**c**) IL-4. (**a–c**) Shown is pooled data from 1–2 independent experiments with 3 biological repeats per experiment (n = 3–6 per group). Error bars represent ± SEM. One-way analysis of variance (ANOVA) with Dunnett’s multiple comparisons test relative to vehicle (control). *P < 0.05; **P < 0.01; ****P < 0.0001.

As BB-Cl-amidine inhibited the induction of Th subsets, we addressed the question of whether already differentiated Th cells, such as those present at the site of inflammation would also be susceptible to BB-Cl-amidine treatment. After differentiating CD4^+^ T cells under Th1, Th17 and Th2 conditions for 5 days, BB-Cl-amidine or vehicle were added for 20 hours. As in the previous experiment in which the drug was added at the start of the culture, there was marked suppression of Th1, Th17 and Th2 cells (Fig. 7) suggesting that Th subsets were still susceptible to the drug even after they had been induced and differentiated. When we examined whether BB-CL-amidine affected T cell survival by inducing apoptosis we found that BB-CL-amidine at 10 μM (high dose) significantly affected cell survival in all culture conditions, with a trend towards increased sensitivity of Th17 cells to PAD inhibition.

## Discussion

In this study, we have shown that the second generation PAD inhibitor, BB-Cl-amidine, reversed joint inflammation both clinically and histologically in collagen-induced arthritis at a dose of 10 mg/kg, with a partial response at 1 mg/kg. Mass spectrometry of proteins identified in lymph nodes, indicate effective inhibition of citrullination *in vivo*, and analysis of serological responses and cytokine production suggested that the profound therapeutic response to the drug was unrelated to the ACPA response, but associated with suppression of pro-inflammatory Th1 and Th17 responses with a relative increase in Th2 responses *in vivo* and *ex-vivo. In vitro* experiments indicated that this was a direct effect of the drug on T helper cells, though, under these conditions all phenotypes were inhibited, including Th2, Th17 and Th1 cells.

The almost complete reversal of arthritis in our study is in contrast to the modest effects seen with the first generation PAD inhibitor, Cl-amidine, reported by Willis e*t al.*^8^. They administered Cl-amidine at similar daily doses (1 mg/kg and 10 mg/kg), with an additional group treated with 50 mg/kg. Unlike our study, they used a prophylactic regimen in which treatment was started immediately after immunisation and continued to day 35, so that at a given dose, each mouse received nearly 4 times as much Cl-amidine compared with the BB-Cl-amidine used in our study. In spite of this much more aggressive treatment regime, the reduction of arthritis was in the region of 50%, and with an approximately 30% improvement in histology scores. Cl-amidine has also been used in mouse models of lupus^7^, inflammatory bowel disease^11^ and cardiovascular disease^12^, with similar profiles of partial therapeutic responses.

The available data involving Cl-amidine suggest that most of its therapeutic effect is due to inhibition of PAD4. In the study from Willis e*t al.*^8^ the dose response to Cl-amidine plateaued at 10 mg/kg/day with no further improvement with 50 mg/kg. Furthermore, in unpublished work available on line^13^ Willis showed that GSK283, a PAD4-specific inhibitor, gave a very similar therapeutic profile. A contribution of PAD4 to the severity of arthritis in other mouse models is suggested by a partial amelioration of the disease in PAD4 knockout mice in TNFα transgenic mice and in mice with glucose-6 isomerase-induced arthritis^14^,^15^. Interestingly PAD4 knockout does not protect against passively-transferred, antibody-mediated arthritis^16^.

We propose that markedly improved therapeutic response to BB-Cl-amidine in CIA is due to its improved pharmacokinetics and its relative potency against PAD2 compared to Cl-amidine, which was documented in detail by Knight *et al*.^7^. They also showed that 1 mg/kg of BB-Cl-amidine was roughly equivalent to 10 mg/kg of Cl-amidine in inhibiting the formation of neutrophil extracellular traps, improving endothelial dysfunction and inhibiting manifestations of murine lupus nephritis^7^. The increased activity of BB-Cl-amidine against PAD2, in addition to its more favourable pharmacokinetic properties, could explain its capability of abrogating arthritis even when treatment is started after the onset of disease. Thus, the development of selective PAD4 inhibitors^17^ for potential therapy in human disease may have been focussing on the wrong isoform of the enzyme.

Using mass spectrometry, citrulline residues were identified in 34 proteins. Noteworthy amongst these was R93 from histone H4, and R117 from histone H3.2. Both are novel citrullination sites on histones and of interest, as R93 is present at the H4/H2B interface and H3 R117 is on the surface of the nucleosomal core particle, close to the DNA^18^. Citrullination of either could result in decondensation of the nucleosome resulting in increased transcription. Interestingly, R117 from histone H3.2 appeared to be citrullinated more in the vehicle-treated arthritic mice and contrary to expectations, citrullination of R93 from histone H4 was only detected in the BB-Cl-amidine treated animals. However, numbers are too small to conclude that the differences resulted from *in vivo* treatment. Future studies will have to address the question of the role of citrullination at both of these sites and their potential influence on gene transcription.

Whilst it is important to appreciate that mass spectrometry is not quantitative^19^, the 90% reduction in the number of citrullinated residues detected in the group treated with 10 mg/kg BB-Cl-amidine would suggest that the drug does effectively deplete citrullinated proteins *in vivo*. To examine the ACPA response in these mice, we chose sequences from α-enolase and vimentin that were highly conserved between mice and humans. Both had arginine-containing controls, and both had been previously tested in murine systems^4^ (and unpublished our observations). Using these peptides, we demonstrated a non-citrulline-specific antibody response to both peptides in the collagen immunised mice compared to naive controls which in the case of vimentin was suppressed by the higher treatment dose. The lack of ACPA in our mice is entirely consistent with the classic studies of Vossennar *et al*.^20^. They examined several mouse models of experimental arthritis and found that several, including CIA, had antibodies detected by first generation CCP assay based on citrullinated sequences from filaggrin. However, when tested with the arginine control peptides, they found equal levels of antibody binding and concluded that this population of anti-CCP antibodies, unlike those found in human RA, where not true ACPA. Other studies have found citrulline-specific antibodies late in disease and these have been attributed to “epitope spreading”. However, these ACPA are at very low levels, compared to the anti-collagen response, and, in general, have only been cited as possibly having an amplifying effect^8^ rather than being the primary mechanism of joint destruction in CIA. In our mice, the experiment was terminated after only 10 days after the onset of arthritis and the response occurred within 5–6 days of treatment. This rapid response together with our serological data would suggest that inhibition of citrullination of autoantigens is an unlikely mechanism for the rapid and profound therapeutic effect of BB-Cl-amidine.

Our study suggests that the response to treatment with BB-Cl-amidine is due to suppression of Th1 and Th17 responses associated with a relative induction of a Th2 type response. Th17 cells are regarded as the most pathogenic subset of T helper cells in chronic arthritis and were identified in this study by their co-expression of RORγT, the Th17 master transcription factor, and IL-17A^21^,^22^. IL-17A increases pro-inflammatory cytokine secretion and recruits monocytes and neutrophils to sites of inflammation^23^,^24^,^25^ and plays a significant pathogenic role in CIA^21^,^26^,^27^,^28^. This indicates that treatment with BB-Cl-amidine is not simply immunosuppressive but immunoregulatory because it also favoured the anti-inflammatory effect of Th2 cells. However, lymph nodes contain factors other than T cells which could be modulated by PAD inhibition. These include monocytes and stromal proteins which could themselves affect the activity of T cells. Therefore, to confirm that the effect of BB-Cl-amidine on T helper subset was direct, we went on to demonstrate that BB-Cl-amidine incubated with Th17 cells induced from naïve (unimmunised mice) suppressed IL-17A production in a dose-dependent manner. There was a similar but less marked effect on IFN-γ production by Th1 cells, and IL-5 production by Th2 cells. This, in turn, suggests that the therapeutic effect of BB-Cl-amidine is predominantly at the transcriptional level though selectivity for the different T helper subtypes seen *in vivo* was less well demonstrated *in vitro.* However analysis of the subtype cell numbers and the number of apoptotic cells suggest that Th17 cells are more sensitive to PAD inhibition than other T helper cell types. Much of the selective effects on Th2 subsets observed *in vivo*, could well be indirect, perhaps due to changes in antigen presenting cells. These cell types are known to be rich in PAD2 and PAD4^29^,^30^ and are also capable of directing T helper subsets. Antigen presenting cells are abundant in lymph nodes *in vivo*, but they were not present in our T helper cell cultures *in vitro*.

Our data suggest that pan-PAD inhibitors could become a new class of targeted small molecular weight compounds with a potential role in the treatment of a variety of IL-17A driven diseases. These include the spondyloarthropathies and inflammatory bowel disease which are known to respond to anti-IL-17 antibody treatment^31^. PAD inhibition may have an additional benefit in treating humans with RA, a disease driven by pathogenic autoantibodies to citrullinated proteins. Therefore in RA, PAD inhibition could not only suppress disease by its immunoregulatory effects, but also by inhibiting the formation of the citrullinated proteins that drive the autoantibody response. There is currently great interest in the therapeutic effect of the induction Tregs by tolerizing protocols^32^,^33^,^34^ and the administration of GM-CSF^35^,^36^ in mice with CIA. In our study Tregs were unaffected by BB-Cl-amidine. Therefore, we would predict that combination treatment aimed to induce Tregs along with PAD inhibition might be synergistic in the treatment of RA whilst also minimising potential toxicity. Whichever approach is taken, we now propose that PAD inhibition, alone, or in combination with other drugs, is a promising approach to treating chronic inflammatory disease in humans.

## Methods

### Induction and assessment of arthritis

All animal experiments were approved by the Clinical Medicine Animal Welfare Ethical Review Board and the UK Home Office and conducted in accordance with the approved guidelines. Arthritis was induced in DBA/1 mice as described previously^6^. Ten to 12-week-old male DBA/1 mice (Harlan) received one subcutaneous 100 μL injection of 200 μg bovine type II collagen in complete Freund’s adjuvant (BD Biosciences) at the base of the tail and on the flank. Post-immunization the mice were monitored daily and once an animal showed signs of arthritis it was randomly assigned to a treatment group. Disease severity was assessed daily by an experienced investigator. Arthritis severity was scored as follows: 0 = normal, 1 = slight swelling and/or erythema, 2 = pronounced swelling, 3 = ankylosis. All four limbs were scored, giving a maximum possible score of 12 per animal. Hind paw swelling was measured daily with calipers (Krœplin).

### BB-Cl-amidine

BB-Cl-amidine was synthesised as described^7^ and given as daily intraperitoneal doses of 1 mg/kg or 10 mg/kg or vehicle (5% DMSO, 10% cyclodextran in H_2_0). All procedures were approved by the Clinical Medicine Animal Welfare Ethical Review Board and the UK Home Office.

### Histological analysis of arthritic paws

Hind paws of animals with CIA on day 10 were fixed in paraformaldehyde, decalcified with EDTA, and paraffin-embedded sections were stained with haematoxylin and eosin. Each joint was scored as follows: 0 = normal; 1 = cell infiltration with no signs of joint erosion; 2 = inflammation with the presence or erosions limited to discrete foci; and 3 = severe and extensive joint erosion with loss of architecture. Three hind paw joints (proximal interphalangeal, metatarsal phalangeal and tarsal-metatarsal) were scored and a mean calculated, giving a maximum possible score of 3 per animal. The histological scoring was conducted by an experienced blinded to the study investigator.

### Cytokine detection

Cytokines were detected in serum using the Meso Scale Discovery platform according to manufacturer’s instructions.

### Antibody measurements

Serum levels of bovine anti–type II collagen–specific IgG1 and IgG2a were measured by ELISA as previously described^37^,^38^. Pooled serum from vehicle-treated mice was used as a standard. To measure anti-citrullinated protein antibodies (ACPA) we used peptides from citrullinated α-enolase (CEP-1; amino acids 4–21: KIHA-Cit-EIFDS-Cit-GNPTVE) and citrullinated (Cit) vimentin cVim (amino acids 60–75: VYAT-Cit-SSAV-Cit-L-Cit-SSVP), together with arginine-containing controls for each peptide, REP-1 and Vim respectively^9^.

### Analysis of T-cell responses

On day 10 after disease onset, the animals were culled and a single-cell suspension prepared from inguinal lymph nodes. 2 × 10^5^ cells per well were cultured in a 96-well plate in 200 μL RPMI-1640 with L-glutamine, 10% fetal calf serum, and penicillin/streptomycin (all from Life Technologies).

For immunophenotyping, cells were stimulated with 0.02 μg/mL PMA, 0.4 μM ionomycin and 1.25 μg/mL Brefeldin A (all from Sigma-Aldrich) for 4 hours. The antibodies used for surface and intracellular staining are listed in Supplementary Table 2. The gating strategy used to identify CD4^+^ T cell subpopulations is described in Supplementary Figure 1. The samples, with the exception of samples in which IL-4 was detected, were fixed and permeabilized with the buffers provided with the FoxP3 Staining Buffer Set (eBioscience). IL-4^+^ T cells were fixed with a 2% solution of formaldehyde (Merck) in PBS. The permeabilization buffer contained: 0.05% saponin, 0.5% BSA (both from Sigma-Aldrich), 2 mM EDTA (Invitrogen), 0.02% sodium azide (G-Biosciences) and PBS. Data was acquired on a CANTO II flow cytometer using FACSDIVA software (all BD Biosciences) and analysed using FlowJo software.

For measurement of proliferative responses, inguinal lymph node cells were cultured for 48 hours in the presence of 0.1 μg/ml anti-CD3ε antibody (clone: 145-2C11) (eBioscience). The thymidine analog, 5-bromo-2-deoxyuridine (BrdU, 50 μM), was added for 18 hours to access cell proliferation. BrdU staining was carried out according to manufacturer’s protocols with an anti-BrdU conjugated antibody (clone B44) (BD Biosciences).

### Mouse T helper differentiation assays

Naïve CD4^+^ T cells were isolated from healthy non-immunised BALB/c mice (The Jackson Laboratory) using a CD4^+^ CD62L^+^ T Cell Isolation Kit (Miltenyi). Naïve CD4^+^ T were cultured in Iscove’s Modified Dulbecco’s Medium with Knockout Serum Replacement (Th17) or RPMI-1640 with 10% fetal calf serum (all from Invitrogen) for 5 days in the presence of Th1 (12 ng/mL IL-12 and 10 μg/mL anti-IL-4), Th2 (40 ng/mL IL-2, 40 ng/mL IL-4 and 10 μg/mL anti-IFN-γ) or Th17 (3 ng/mL TGF-β, 30 ng/mL IL-6, 10 ng/mL IL-1β, 10 ng/mL IL-23, 10 μg/mL anti-IL-4 and 10 μg/mL anti-IFN-γ) conditions and anti-mouse CD3 (1.0 μg/mL; clone 145-2C11, eBioscience) and anti-mouse CD28 (0.5 μg/mL; clone 37.51, eBioscience). Neutralising antibodies were supplied from BioLegend. All cytokines were supplied by PeproTech, except IL-23 (R&D Systems).

### Mass spectrometric analysis of proteins

Proteins were precipitated from cells using chloroform/methanol followed by proteolysis with trypsin^39^. Peptides were desalted and analysed as described previously^19^. Data was analysed with PEAKS^19^. Spectra representing citrullinated peptides were manually inspected for mono-isotopic precursor and fragment annotation to exclude false positives.

### Apoptosis detection

Dead and apoptotic cells were detected using the Annexin-V Apoptosis Detection Kit along with propidium iodide according to manufacturer’s instructions (Sigma-Aldrich).

### Statistical analysis

Data are presented as the arithmetic mean ± standard error of mean (SEM). Comparisons between groups were carried out using an analysis of variance (ANOVA) with Dunnett’s (one-way) or Tukey’s (two-way) post hoc test. Probability values (*P*) of less than 0.05 were considered significant. For the animal studies (collagen-induced arthritis, CIA), >8 mice were randomly selected per group. This was based on the calculation that a sample size of 8 per group would have a 90% power to detect a difference between a mean clinical score of 2 with a significance level (alpha) of 0.05 (two-tailed). The *in vivo* CIA experiments were repeated twice to ensure reproducibility and then the data pooled together. For the *in vitro* studies, experiments were conducted with at least 3 biological repeats. The experiments were repeated two to three times to ensure reproducibility and then the data pooled together. Statistical analysis was performed using GraphPad PRISM (Graphpad Software Inc., USA).

## Additional Information

**How to cite this article**: Kawalkowska, J. *et al*. Abrogation of collagen-induced arthritis by a peptidyl arginine deiminase inhibitor is associated with modulation of T cell-mediated immune responses. *Sci. Rep.* **6**, 26430; doi: 10.1038/srep26430 (2016).

## Supplementary Material

Supplementary Information
