# Supplementary material for: Abrogation of collagen-induced arthritis by a peptidyl arginine deiminase inhibitor is associated with modulation of T cell-mediated immune responses
Source: Sci Rep. 2016 May 23;6:26430. doi: 10.1038/srep26430 (PMC4876390; doi:10.1038/srep26430)
Supplement: Supplementary Information [file srep26430-s1.pdf]

Abrogation of collagen-induced arthritis by a peptidyl arginine deiminase inhibitor is associated with modulation of T cell-mediated immune responses

Joanna Kawalkowska[1*], Anne-Marie Quirke[1*], Fatemeh Ghari[2], Simon Davis[4], Venkataraman Subramanian[3], Paul R. Thompson[3], Richard O. Williams[1], Roman Fischer[4], Nicholas B. La Thangue[2] and Patrick J. Venables[1]

[1]Kennedy Institute, Nuffield Department of Orthopaedics, Rheumatology & Musculoskeletal Sciences, University of Oxford, Roosevelt Drive, Oxford OX3 7FY, UK

[2]Laboratory of Cancer Biology, Department of Oncology, University of Oxford, Old Road Campus Research Building, Roosevelt Drive, Oxford OX3 7DQ, UK

[3]Biochemistry and Molecular Pharmacology, University of Massachusetts Medical School, LRB 826, 364 Plantation Street, Worcester, MA, 01605, USA

[4]Target Discovery Institute, Nuffield Department of Medicine, University of Oxford, Roosevelt Drive, Oxford OX3 7FZ, UK

* Contributed equally to the study as joint first authors

SUPPLEMENTARY FIGURE 1

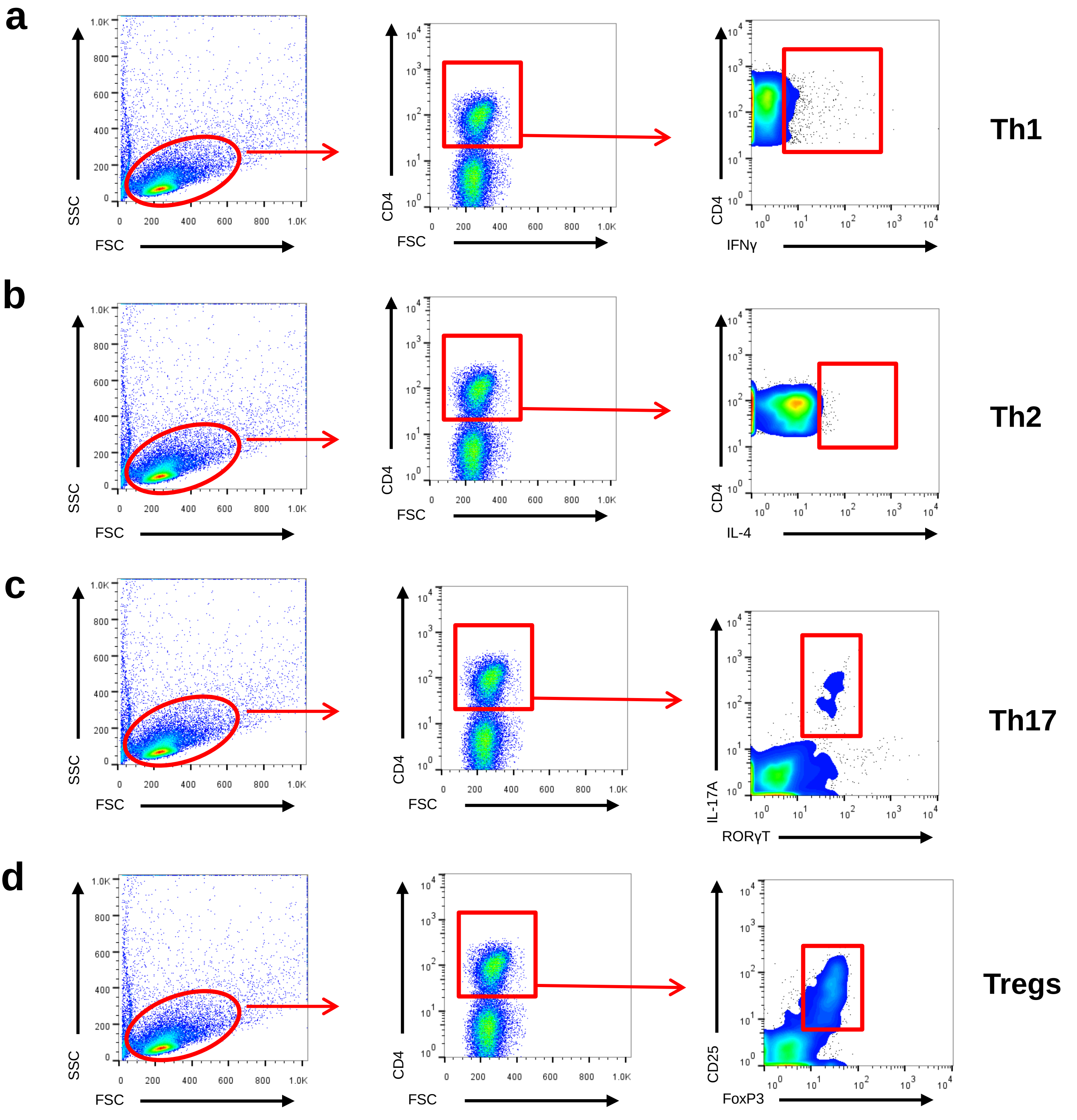

The gating strategies used in flow cytometry to identify CD4⁺ T cell subpopulations in the lymph nodes of arthritic mice

(**a-c**) T helper subsets were identified with antibodies targeted against CD4 and IFN-γ (Th1), IL-4 (Th2) or IL-17A (Th17). (**d**) Tregs were designated as CD4⁺ CD25⁺ FoxP3⁺.

SUPPLEMENTARY TABLE 1

Mass spectrometry analysis of citrulline residues in proteins isolated from the lymph nodes of mice with collagen-induced arthritis. The number of mice (out of 5-6 mice per group) which had citrulline detected in that protein is shown.

Protein	Accession	Site	Vehicle	1 mg BB-Cl	10 mg BB-Cl	Naïve
Prothrombin	H7BX99	R490	3	2		
Actin, , cytoplasmic 1	P60710	R290	2	1		
Elongation factor 1-alpha	P10126	R266	2	1		
RNA demethylase ALKB5	Q3TSG4	R287	2			
Histone H4	P62806	R93	1	1	2	
Histone H3.2	P84228	R117	1	1		
YLP motif containing protein	F6YTL8	R579	1	2		
Actin-related protein 2	P61161	R319	1			
14-3-3 protein epsilon	P62259	R19	1			
Gelsolin	P13020	R449	1			
Probable ATP-dependent RNA helicase	Q80Y44	R478	1			
Keratin type 1 cytoslkeletal17	Q9OWL7	R410	1			
Myosin-9	Q8VDD5	R143	1			
Beta-globin	A8DUK4	R410	1			
Probable ATP-dependent RNA helicase	Q61656	R2523	1			
Spondin-1	Q8VCC9	R93	1			
Acyl-CoA dehydrogenase 10	Q8K370	R456	1			
Collagen alpha-1(VII)	Q63870	R2523		1	1	
Tropomyosin alpha 3 chain	D3Z6I8	R124		1		
Trifunctional enzyme subunit alpha	Q8BMS1	R610		1		
Fat1	F2Z4A3	R3775		1		
Fatty acid synthase	P19096	R2019		1		
Histone deacetylase 1	O09106	R229		1		
RNA demethylase ALKBH5	Q3TSG4	R287		1		
Elongation factor 1-alpha	P10126	R134		1		
Inter-alpha-trypsin inhibitor heavy ITIH2	P19823	R341		1		
Inter-alpha-trypsin inhibitor heavy ITIH3	Q06033	R70		1		
Heterogeneous nuclear ribonuclearprotein A3	Q8BG05	R68		1		
Keratin, type2 cytoskeletal 1b	Q6IFZ6	R342		1		
Keratin, type2 cytoskeletal 75	Q8BGZ7	R239		1		
Beta-2-glycoprotein 1	Q01339	R139		1		
Treacle protein	O08784	R1068			1	
Serin/arginine-rich splicing factor 5	O35326	R48			1	

SUPPLEMENTARY TABLE 2

The antibodies used in flow cytometry to identify $CD4^+$ T cell subpopulations in the lymph nodes of arthritic mice and in T cell cultures

**Antibody**	**Clone**	**Manufacturer**
CD4	GK1.5	eBioscience
CD25	PC61.5	eBioscience
IL-17A	eBiol17B7	eBioscience
RORγT	Q31-378	BD Biosciences
IFN-γ	XMG1.2	eBioscience
IL-4	11B11	eBioscience
FoxP3	FJK-16s	eBioscience
GATA3	TWAJ	eBioscience
